# Salivary Biomarkers for Oral Cancer Detection: An Exploratory Systematic Review

**DOI:** 10.3390/ijms25052634

**Published:** 2024-02-23

**Authors:** Daniel Bastías, Alejandro Maturana, Constanza Marín, René Martínez, Sven Eric Niklander

**Affiliations:** Unit of Oral Pathology and Oral Medicine, Faculty of Dentistry, Universidad Andres Bello, Viña del Mar 2520000, Chileconstanza.marin@unab.cl (C.M.)

**Keywords:** mouth neoplasm, oral cancer, oral potentially malignant disorder, biomarkers, saliva

## Abstract

Different efforts have been made to find better and less invasive methods for the diagnosis and prediction of oral cancer, such as the study of saliva as a source of biomarkers. The aim of this study was to perform a scoping review about salivary molecules that have been assessed as possible biomarkers for the diagnosis of oral squamous cell carcinoma (OSCC). A search was conducted using EBSCO, PubMed (MEDLINE), Scopus, and Web of Science. The research question was as follows: which molecules present in saliva have utility to be used as biomarkers for the early detection of oral cancer? Sixty-two studies were included. Over 100 molecules were assessed. Most of the markers were oriented towards the early diagnosis of OSCC and were classified based on their ability for detecting OSCC and oral potentially malignant disorders (OPMDs), OSCC outcome prediction, and the prediction of the malignant transformation of OPMDs. TNF-α, IL-1β, IL-6 IL-8, LDH, and MMP-9 were the most studied, with almost all studies reporting high sensitivity and specificity values. TNF-α, IL-1β, IL-6 IL-8, LDH, and MMP-9 are the most promising salivary biomarkers. However, more studies with larger cohorts are needed before translating the use of these biomarkers to clinical settings.

## 1. Introduction

Oral squamous cell carcinoma (OSCC) is the most common form of head and neck cancer [1]. It can arise de novo or from oral precursor lesions, a group known as oral potentially malignant disorders (OPMDs) [2]. OPMDs encompass different disorders, with oral leukoplakia (OL) being the most common [3], with an estimated annual malignant transformation rate of 10% [4]. Despite different technological advances in the medical sciences, is still not possible to predict which OPMD will progress into cancer [5,6], with clinicians and pathologists still relying on the same histopathological and clinical indicators used for the last 40 years. There are also significant challenges for diagnosing oral cancer in early stages. Despite the oral cavity being of “easy access”, OSCC is still being diagnosed mostly in stages III and IV of the disease, which is translated to significant morbidity and an estimated overall survival of ≈50% during the first 5 years [7]. 

Different efforts have been made to discover biomarkers to aid in the diagnosis of OPMDs and OSCC in order to predict the malignant transformation of precursor lesions, treatment response, the development of regional and distant metastases, among others [8]. Biomarkers have been obtained from tissues, urine, serum, blood, and saliva, among others. Saliva is probably the most attractive secretion for the finding of biomarkers for OSCC and OPMDs, as its collection is easy, non-invasive, fast, and cost-effective. Thus, different studies have explored the possibility of using salivary factors as biomarkers for the development and progression of OSCC and OPMDs [9,10,11]. The aim of this study was to perform an exploratory systematic review about the different salivary molecules that have been assessed as possible biomarkers for the diagnosis of OSCC.

## 2. Materials and Methods

### 2.1. Study Design

This scoping review was performed according to the Preferred Reporting Items for Systematic Reviews and Meta-Analysis (PRISMA) guidelines and scoping review guidelines from the Joanna Briggs Institute [12]. The research question was as follows: which molecules present in saliva have utility to be used as biomarkers for the early detection of oral cancer?

### 2.2. Inclusion and Exclusion Criteria

Inclusion criteria were as follows: full text articles in English published between January 2015 and July 2022; studies describing any salivary molecule used as biomarker for the detection of oral cancer or an oral potentially malignant disorder; and clinical trials and cohort and case–control studies involving humans.

Exclusion criteria were as follows: reviews, editorials, opinions, letters to the editor, case reports, animal studies, studies about salivary biomarkers of other cancers, in vitro cell line studies, and animal studies.

### 2.3. Information Sources and Search Strategy 

A bibliographic search was conducted using EBSCO, PubMed (MEDLINE), Scopus, and Web of Science with a publication date ranging from January 2015 to July 2022.

The following MeSH (Medical Subject Headings) search terms were used: “Saliva”, “Biomarker”, “Diagnosis”, “Head and neck cancer”, “Mouth neoplasms”, “Oral Cancer”, and “Precancerous conditions”, which were combined using “AND” or “OR”. Additionally, a manual search was also performed (Supplementary Figure S1).

### 2.4. Selection Process

Following the literature search, the records from each database were exported into Mendeley refence manager (Elsevier, London, UK, 2024). Duplicates were removed at this stage. The selection of articles was conducted independently by two reviewers (A.M. and D.B.). In the first phase, both reviewers independently screened the titles and abstracts for relevance. Any disagreements were resolved by S.N. In the second phase, both reviewers independently performed a full-text review (A.M. and D.B.). Disagreements at this stage were resolved by S.N.

### 2.5. Data Extraction

The following data were extracted into a spreadsheet (Office Excel 2016; Microsoft Corp., Redmond, WA, USA): author, year of publication, country, diagnosis, number of cases included, biomarker, technique, and statistical analysis employed.

## 3. Results

**Figure 1:**
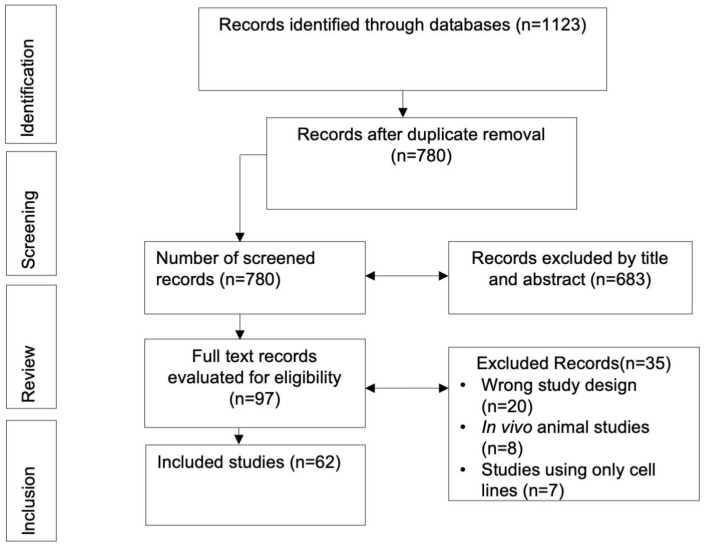
PRISMA flow chart of selected studies.

The systematic search initially retrieved 1123 records. After eliminating 343 duplicates, 780 articles were subjected to title and abstract review, resulting in 97 records for full-text evaluation. Of those, 35 were excluded due to not meeting the inclusion criteria; thus, 62 articles were included for data analysis (Figure 1).

### 3.1. Characteristics of the Included Studies 

The studies included the analysis of over 60 molecules present in the saliva of 5278 patients, of which 2546 were patients with OSCC, 1070 with an OPMD, and 1662 healthy controls. The range of patients included per study varied from 23 to 310, and the range of biomarkers ranged from 1 to 11 (Supplementary Table S1). 

Most of the detected markers were oriented towards the early diagnosis of OSCC (n = 54 molecules) and were classified based on their ability for detecting OSCC and OPMDs, OSCC outcome prediction (n = 4 molecules), and predicting the malignant transformation of OPMDs (n = 6 molecules). A list of abbreviations of the different markers can be found at the end of the manuscript. 

### 3.2. Biomarkers for the Detection of OSCC and OPMDs 

#### 3.2.1. Enzymes

Eight articles assessed salivary metalloproteinase (MMP) levels, with MMP-9 being the most frequently studied. One study reported statistically significant differences in salivary MMP-9 and MMP-2 concentrations between OPMD patients compared to controls (*p* = 0.05 and *p* = 0.02, respectively) [13]. Another study reported significantly higher salivary MMP9 levels (*p* < 0.001) in OSCC patients compared to OPMD patients, with an AUC of 0.917 for OSCC and an AUC of 0.852 for OPMD [14]. Ghallab and Shaker assessed the ability of MMP9 salivary levels to differentiate between patients with OSCC and an OPMD, reporting a sensitivity and specificity of 100% [15]. Other MMPs have also been reported to be altered between healthy and OSCC patients [16,17]. (Supplementary Table S2).

Other enzymes, such as LDH, AKR1b10, l-fructose, cathepsin V, and kallikrein, have shown elevated salivary concentrations in OSCC patients when compared to control groups [18,19,20,21,22,23]. Among these six markers, LDH is the most studied enzyme.

A significant rise in LDH levels in OSCC and high-risk premalignant lesions compared to control groups was reported [18], with a 3.9-fold and 2.5-fold increase, respectively. Similar results have also been reported by others [22]. The average salivary levels of AKR1b10 were significantly higher in patients with OSCC (646.47 ± 402.43 pg/mL) compared to healthy controls (25.0.1 ± 32.96 pg/mL), and OSCC patients with salivary AKR1b10 values over 646 pg/mL had significantly lower survival rates compared to OSCC patients with lower AKR1b10 levels [21]. Other enzymes and proteases, including kallikrein 5, cathepsin V, and ADAM9, also showed a significant increase in the saliva of patients with OSCC, each presenting an AUC > 0.7. The combination of these three markers improved diagnostic accuracy, with an AUC > 0.9 [19] (Supplementary Table S2).

#### 3.2.2. Glycoproteins

CEA and CD44 are both included in this category [24,25,26,27,28,29]. CEA was the subject of study in three studies, and in all of them, a significant increase in the saliva of patients with OSCC compared to control patients was reported (*p* < 0.005) [22–25.52]. CEA salivary levels were also analyzed in conjunction with another marker, Naa10p, resulting in a significant increase in sensitivity and specificity (92.5% and 85%) [27]. CD44 variants were also found to be increased in the saliva of OSCC compared to control patients. A statistical analysis suggested a probable role of CD44v6 in the early stages of malignancies and of both CD44v6 and CD44v10 in locoregional aggressiveness and histopathological conditions [26] (Supplementary Table S2).

#### 3.2.3. Cytokines

Several studies analyzed the salivary expression of different cytokines, including IL-4, IL-6, IL-10, IL-13, IL-1β, IL-1RA, IL-17A, IL-17F, IFN-γ, TNF-α, HGF, CRP and VEGF [30,31]. The most studied cytokine was IL-8. In all these studies, a significant increase in IL-8 in the saliva of OSCC patients compared to controls was reported (*p* < 0.05) [32,33,34,35,36,37,38,39]. The diagnostic capacity of IL-8 was also evaluated in combination with a panel of different markers, including DUSP1, H3F3A, and SAT. This combination resulted in an improvement in diagnostic accuracy with an AUC of 0.77 [33]. Another study reported both IL-8 and IL-1β to be effective salivary diagnostic markers in all stages of OSCC, showing significant discrimination, especially in stages III and IV and postoperative phases of the disease (*p* < 0.05) [37]. Similar results were also reported for IL-17A and IL-17F, which were significantly overexpressed in the saliva of patients with more advanced stages of the disease (*p* < 0.001 and *p* < 0.01, respectively), particularly in stage IV. Furthermore, patients with larger primary tumors (T4) and greater nodal involvement were also found to have higher levels of IL-17 [40]. TNF-α was also studied in a significant number of studies [32,33,35,40,41,42]. TNF-α concentrations in the saliva of OSCC [35] and OPMD patients [41] have been reported to be significantly higher than in control patients, with an AUC > 0.8 for both OSCC and OPMD [32,33]. Other cytokines such as HGF and VEGF have also been reported to be significantly higher in the saliva of OSCC patients [43] (Supplementary Table S2). 

#### 3.2.4. MicroRNAs

Several studies have investigated the concentrations of different MicroRNAs in patients with OSCC and OPMDs. miR-21, miR-184, miR-let-7-5p, miR-412-3p, miR-512-3p, miR-302-3p, miR-517-3p, miR-30c-5p, miR-SAT, miR-OAZ, miR-H3F3A, and miR-24-3p have all shown significant differences in patients with OSCC and/or an OPMD compared to control groups [44,45,46,47,48,49]. Care has to be taken when assessing salivary RNA levels as, according to one study, these might be affected by periodontal status [50] (Supplementary Table S2). 

#### 3.2.5. Metabolites

Five studies explored the use of metabolites as saliva biomarkers for the diagnosis of OSCC and OPMDs. One study selected three metabolites (ornithine, ohydroxyvenzonate, and R5P) to assess their ability to distinguish between patients with OSCC and oral dysplasia, obtaining an AUC of 0.871 [51] (Supplementary Table S2). Two other studies examined salivary concentrations of TSA and compared them among patients with OSCC with different grades of differentiation (mild, moderate, and severe). In both studies, a significant increase in TSA was observed in the saliva of patients with less differentiated tumors (*p* < 0.01) [50,52]. Glycine and proline were also reported to be significantly elevated in the saliva of patients with OSCC (*p* = 0.02) [53] (Supplementary Table S2).

#### 3.2.6. Others

ANG, ANG2, NUS1, transgelin and RCN1, showed significant increases in patients with OSCC compared to healthy controls [43,54,55,56]. Cai et al. reported a 1.58-fold increase in ANG and a 13.56-fold increase in ANG2 in OSCC patients [43]. Regarding the utility of NUS1 and RCN1, Ueda et al. reported a sensitivity of 68% and 70%, respectively, which increased to 98% when combining both markers [54]. Another study analyzed the concentrations of FSA and PBSA in OSCC patients who use tobacco, non-cancer patients who chew tobacco, and healthy controls without tobacco use. Significant increases in the concentrations of both markers were recorded among patients with OSCC and tobacco users [57]. KPNA2 has been proposed as a marker for disease progression, as a study that analyzed its salivary levels in OSCC patients in different stages of the disease found a significant increase in advanced stages (III and IV) and a positive correlation with lymph node spread [58]. In a similar way, a recent study that analyzed salivary concentrations of LGALS3BP reported a discriminative concentration of that marker for OSCC in stages I and II and high-risk OPMDs, but a decrease in the late stages and postoperative recurrences [37]. Vitamin C, L-fructose, CYFRA21-1, β2-microglobulin, Cathepsin B and the expression of many genes coding for different proteins involved in different functions have also been reported to be altered in OSCC and OPMD patients [59,60,61,62,63,64,65,66,67] (Supplementary Table S2).

### 3.3. Biomarkers for OSCC Outcome Prediction

Several markers have been proposed for this purpose, including IL-5, CYFRA-21, miR-139-5p, and EGFR [38,53,68,69,70]. Salivary miR-139-5p levels were able to discriminate between patients with OSCC before surgical treatment from healthy individuals (AUC = 0.805) and from patients with OSCC after surgery (AUC = 0.713). Furthermore, concentrations of this marker returned to normal in the postoperative period (4–6 weeks) in patients undergoing tumor excision [68]. Similarly, high CYFRA 21-1 salivary levels have been associated with OSCC recurrence (*p* = 0.0031) [69] and high salivary levels of EGFR with poor survival [70]. Another study found an increase in salivary IL-5 levels after the first surgery. This was followed by a decrease in IL-5 in patients where a second primary occurred, which was followed by an increase in IL-5 saliva concentrations after the tumor was removed [38].

### 3.4. Biomarkers for the Prediction of Malignant Transformation of OPMDs

Certain salivary biomarkers have been assessed to try to predict the malignant transformation of OPMDs. Zaharan et al. reported an increase in salivary miRNA-21 and miRNA-184 in OPMDs (with and without dysplasia) and OSCC when compared to healthy and other disease control subjects [48]. Similarly, Sabarathinam et al. reported that salivary GPx, MDA, TNF-α, and AFP gradually increased as progression occurred from OPMDs to OSCC [42]. Another study showed that miRNA-31 increased in OPMDs and OLP with dysplasia and when the malignant transformation of previous an OMPD happened [47].

## 4. Discussion

According to the World Health Organization, in the year 2018 alone, 177,384 deaths due to oral cancer were recorded worldwide [71]. Different efforts have been made to find better and less invasive methods for the diagnosis and prediction of oral cancer, such as the use of saliva as a biomarker source [58], resulting in over 100 markers with potential use in OSCC patients. Nevertheless, there is no worldwide consensus on a unique biomarker (or a combination of them) able to accurately diagnose and predict OSCC development [8].

Among the analyzed markers, some showed more promise than others. IL-5 [38], miRNA-31 [47], among some others, have reported good results and positioned themselves as good candidates. Nevertheless, these were analyzed by only one study (IL-5 and miRNA-31). A similar scenario is reiterative with various markers identified in this review, with fifty-five of them being assessed in only one study. Consequently, it becomes challenging to establish the potential of all these molecules, given that the evidence is very limited, which makes it impossible to draw any conclusions.

The study of miRNA holds promise. Different systematic reviews have also concluded that they are potential biomarkers for OSCC and OPMDs [11,72,73]. The main issue is, as with many of the other markers, is that apart from mir-21, which was assessed by two studies [47,48], the salivary expression of all other miRNAs, has been analyzed by single studies.

However, among all markers, there were six assessed by four or more studies, all reporting high sensitivity and specificity values for the diagnosis of oral squamous cell carcinoma (OSCC) and/or OPMDs. These markers were IL-1β, IL-6, IL-8, LDH, MMP-9, aTNF-α (Table 1).

Interleukin-8 can play different roles in the carcinogenesis process, including the promotion of epithelial-to-mesenchymal transition [38], angiogenesis [35], and tumor growth [34]. This is probably why it was the most frequently investigated biomarker in this review (seven studies). All studies that evaluated IL-8 in OSCC reported its usefulness for discriminating between healthy and oral cancer patients [34,35,37,38]. However, there were discrepancies regarding at which stage of the disease the detection of IL-8 was most relevant. Some studies suggested salivary IL-8 to be useful in early stages, while others associated its increase with late stages of the disease. Apparently, IL-8 is better at discriminating OSCC than OPMDs, as only 60% of the reviewed articles indicated a rise in IL-8 in OPMDs [32,35,37,38]. Nevertheless, care must be taken when interpreting these findings, as two reports pointed out that periodontal disease and tobacco consumption could have affected IL-8 concentrations [34,37]. In contrast, Lee et al. dismissed the idea that factors such as alcohol or tobacco could directly influence salivary concentrations of IL-8, as their results did not demonstrate significant differences between groups associated with these factors when compared to control groups [35].

LDH is a catalyzing enzyme found in most cells, participating in cellular metabolism, playing a key role in the final step of the anaerobic–glycolytic metabolic pathway [18,20,22]. According to this review, salivary concentrations of these enzymes could be related to tumor size and cancer stage. Additionally, there may be potential utility in distinguishing between malignant and potentially malignant conditions, such as leukoplakia or submucosal fibrosis [18,23,75]. These results agree with the role of LDH in the organism. Awasthi suggested that elevated levels of this enzyme could be explained by uncontrolled cell mass increase, leading to the release of free analytes into the saliva [18]. LDH is an enzyme associated with cell membrane damage, and therefore, the quantification of salivary LDH could be related to death or cellular damage [20,22]. Different factors could limit the use of this enzyme as a biomarker, so care must be taken. For example, some authors suggest that salivary LDH content could be affected by various local or systemic factors such as periodontitis or tobacco consumption [22,23]. Nevertheless, all studies that investigated salivary LDH expression propose it as a promising marker in the diagnosis, monitoring, and prognosis of OSCC and/or OPMDs. However, they all agree that studies with a larger population, including patient follow-up, are needed [18,20,22,23,74]. 

IL-1β is constitutively overexpressed in OSCC [76] and is identified as a key node gene in the tumor microenvironment (TME) of OSCC in vivo [77]. The oncogenic properties of IL-1β have also been demonstrated using in vivo models. In a mouse oral cancer model, pIL-1β mRNA positively correlated with the presence of malignant change. IL-1β is also reported to have a role in the induction of EMT in OSCC, as in the same study, OSCC cells treated with IL-1β showed molecular signs of EMT induction [78], which has also been reported by others [79,80,81]. This is also supported by the fact that IL-1β silencing reduces OSCC tumor size in vivo [77] and that elevated IL-1β expression has been related with lymph node metastasis in OSCC [82]. IL-1β salivary levels are able to discriminate between OSCC subjects and controls [35,37,83], but not between OPMD patients and healthy subjects [37]. The reported AUC of salivary IL-1β to differentiate between OSCC and control individuals varies between 0.729 and 0.7724 [35,37], but increases significantly when considering only late-stage OSCC [37]. Importantly, it has been reported that the discriminatory power of salivary IL-1β increases when used with other markers, such as IL-8, SAT1, and DUSP1 [83]. In the study by Singh et al., salivary IL-1β failed to distinguish between post-treatment OSCC individuals and healthy subjects, suggesting the normalization of IL-1β salivary levels after tumor removal [37]. A study in which the authors analyzed the expression of IL-1β (among other 50 factors) in OSCC patients before and after surgical intervention showed a significant decrease in salivary IL-1β levels after tumor resection. No significant changes in other cytokine levels were reported [84]. Similar results were also reported elsewhere [38]. 

MMP9 has been described as having multiple functions, including its participation in protein and extracellular matrix degradation [15], promotion of angiogenesis [15,75] and regulation of the initial steps of carcinogenesis and invasion [14]. Only one out of the four studies that included this molecule in their analysis determined that the differences in salivary concentrations of MMP-9 between patients with OSCC and control subjects were of no significance [15]. Most of the studies agreed on the potential of this biomarker for the diagnosis of OSCC and OPMDs [13,14,15,75], with possible utility for predicting the malignant transformation of previous OPMDs and the treatment monitoring of OSCC [14,51,75]. These results make sense when considering the functions of this metalloproteinase in the organism. MMP-9 maintains the bioavailability of growth factors such as VEGF. Consequently, an increase in MMP-9 would indirectly promote angiogenesis and uncontrolled cell proliferation [13,51,75,85]. 

TNF-α has been reported to be an important molecule within the inflammatory process [33,35,41,42] also capable of stimulating the growth of fibroblasts and inducing cell death [20]. TNF-α was suggested to have possible utility in distinguishing between OPMDs, OSCC, and healthy individuals [33,35,41,42]. TNF-α induces the release of other cytokines, thus creating a tumor microenvironment that favors the proliferation and survival of malignant cells [41]. In this regard, some authors proposed salivary TNF-α to be related to the patient’s lymph node involvement [32,40]. 

IL-6 is considered and oncogenic cytokine, similar to IL-1 and IL-8, and is reported to cause EMT [86], induce angiogenesis and tumor growth [87,88], disrupt cell–cell communication, impede macrophage function, and promote epithelial and endothelial cell migration and invasion [89]. IL-6 overexpression in patients with HNSCC is associated with poor prognosis, probably by enabling an immunosuppressive TME by increasing the presence of myeloid-derived suppressor cells and PDL-1 expression, and is considered a significant predictor of treatment outcome [90]. In OSCC, the expressions of IL-6 and IL-8 are associated with a more invasive mode of growth [91]. Apart from the study from Khyani et al., all studies that assessed IL-6 salivary expression reported an increase in OSCC patients [32,33,35,38].

## 5. Conclusions

Salivary biomarkers have the potential to become a useful tool to aid in the detection, control, and prediction of the malignant transformation and metastasis of OPMDs and OSCC. All authors agree that is unlikely that one single molecule would meet the requirements to be used as a biomarker, so efforts should be focused on developing a panel of biomarkers. TNF-α, IL-1β, IL-6 IL-8, LDH, and MMP-9 are the most promising markers with the strongest evidence. However, in all cases, more studies with larger cohorts and more robust study designs are needed before translating the use of these markers to clinical settings. It would be interesting for future studies to assess the sensitivity and specificity of these markers when combined. 

## Figures and Tables

**Table 1:** Markers studied by 4 or more studies.

Marker	Function	Expression	References
LDH	Diagnosis	Increased	[18,20,22,23,52,74]
TNF-α	Diagnosis, prognosis	Increased	[32,33,35,40,41,42]
MMP-9	Diagnosis, transformation	Increased	[13,14,15,75]
IL-8	Diagnosis	Increased	[32,33,34,35,36,37,38]
IL-1β	Diagnosis, prognosis	Increased	[33,35,36,37,38]
IL-6	Diagnosis Prognosis	Increased	[32,33,34,35,38]

## Supplementary Materials

The following supporting information can be downloaded at https://www.mdpi.com/article/10.3390/ijms25052634/s1.

## Abbreviations

**Abbreviation**	**Name**
AFP	Alpha-Fetoprotein
AKR1B10	Aldo-Keto Reductase Family 1 Member B10
CD44	Cluster of Differentiation 44
CEA	Carcinoembryonic Antigen
CRP	C-Reactive Protein
CSTA	Cystatin A
CYFRA 21-1	Cytokeratin Fragment 21-1
EGFR	Epidermal Growth Factor Receptor
ErbB2	Erb-B2 Receptor Tyrosine Kinase 2 (HER2)
FSA	Fibrin Split Products A
GPx	Glutathione Peroxidase
GRO	Growth-Related Oncogene
HCC-1	Hepatocellular Carcinoma-Associated Protein
HGF	Hepatocyte Growth Factor
IFN-y	Interferon-gamma
IL	Interleukin
KLK5	Kallikrein 5
KPNA2	Karyopherin Alpha 2
LGALS3BP	Galectin-3-Binding Protein
LGALS 3bp0	Galectin-3-Binding Protein (Alternative Name)
LDH	Lactate Dehydrogenase
MCP-1	Monocyte Chemoattractant Protein-1
DUSP1	Dual-Specificity Phosphatase 1
MAOB	Monoamine Oxidase B
MDA	Malondialdehyde
MMP	Matrix Metalloproteinase
mRNA	Messenger RNA
miR	MicroRNA
Naa10p	N-alpha-acetyltransferase 10
NUS1	Nucleolar Complex Protein 1
PADI1	Peptidyl Arginine Deiminase 1
PBSA	Para-Benzenesulfonic Acid
PF-4	Platelet Factor 4
PIGF	Placental Growth Factor
RCN1	Reticulocalbin 1
RSP	Ribosomal Protein S
SAT	Spermidine/Spermine N1-Acetyltransferase
SYNE1	Spectrin Repeat Containing Nuclear Envelope Protein 1
S100	S100 Calcium-Binding Protein
SLC3A2	Solute Carrier Family 3 Member 2
TIMP	Tissue Inhibitor of Metalloproteinases
TNC	Tenascin C
TNF-α	Tumor Necrosis Factor Alpha
TSA	Tumor-Specific Antigen
VEGF	Vascular Endothelial Growth Factor
8-OHdG	8-Hydroxy-2’-deoxyguanosine
