# Supplementary material for: Salivary Biomarkers for Oral Cancer Detection: An Exploratory Systematic Review
Source: Int J Mol Sci. 2024 Feb 23;25(5):2634. doi: 10.3390/ijms25052634 (PMC10932009; doi:10.3390/ijms25052634)
Supplement: Supplementary file 1 [file ijms-25-02634-s001.zip › Supplementary Figure S1.pdf]

Search strategy for each database

**WOS**<br>(((((ALL=(saliva)) AND ALL=(diagnosis)) AND ALL=(biomarker)) AND ALL=(oral cancer)) OR ALL=(head and neck cancer)) OR ALL=(premalignant lesion)

**Scopus**<br>saliva AND biomarker AND diagnosis AND oral AND cancer OR head AND neck AND cancer OR premalignant AND lesion

**Pubmed (Medline)**<br>(((((Saliva) AND (diagnosis)) AND (biomarkers)) AND (mouth neoplasm)) OR (head and neck neoplasm)) OR (precancerous condition)

**Ebsco**<br>saliva AND diagnosis AND biomarker AND precancerous lesion OR oral cancer OR (head and neck cancer)
