# Supplementary material for: Salivary Biomarkers for Oral Cancer Detection: An Exploratory Systematic Review
Source: Int J Mol Sci. 2024 Feb 23;25(5):2634. doi: 10.3390/ijms25052634 (PMC10932009; doi:10.3390/ijms25052634)
Supplement: Supplementary file 1 [file ijms-25-02634-s001.zip › Supplementary tables .pdf]

Supplementary Table S1. Biomarkers and disorders analyzed by the different studies included in this review.

Reference	Groups	Sample size	Biomarkers
[44]	OSCC (20)<br>OPMD (60)<br>CONTROLS (20)	100	miR-21
			miR-184
			miR-145
[43]	OPDM (46)<br>CONTROLS (24)	68	miR-21
			miR-31
[50]	OSCC (50)<br>CONTROLS (50)	100	Cyfra-21
[30]	OSCC (54)<br>CONTROLS (31)	85	SAT mRNA
			OAZ mRNA
			IL-1β mRNA
			IL-8 mRNA
[31]	OSCC(58)<br>OPMD (47)<br>CONTROLS (42)	117	IL-1β
			IL-8
			LGALS3BP
[40]	OSCC (150)<br>CONTROLS (80)	230	miR-let-7a-5p
			miR-3928
			miR-7703
			miR345-5p
			miR1470

[52]	OSCC (20)<br>CONTROLS (20)	40	CD44
[34]	OSCC (30)<br>OPMD (30)<br>CONTROLS (30)	90	TNF-α
[16]	OSCC (30)<br>OPMD (9)<br>CONTROLS (25)	64	CYFRA 21-1
			LDH
			Amylase
[53]	OSCC (30)<br>CONTROLS (33)	63	IL-4
			IL-10
			IL-13
			IL-1RA
[35]	OPMD (10)<br>OSCC (15)<br>CONTROLS (15)	35	GPx
			MDA
			TNF-α
			AFP
[54]	OSCC (78)	78	Transgeline mRNA
[18]	OSCC (25)<br>OPMD (25)<br>CONTROLS (10)	60	LDH
[27]	OSCC (31)<br>CONTROLS (59)	90	H3F3A mRNA
			IL-8 mRNA
			IL-1β mRNA
			DUSP1 mRNA
			OAZ1 mRNA
			SAT1 mRNA
			S100P mRNA
			TNF-α mRNA
			IL-6 mRNA

[55]	OSCC (16)<br>CONTROLS (5)	21	N-glycan
[45]	OSCC (14)<br>CONTROLS (10)	24	TSA
[49]	OSCC (25)<br>CONTROLS (25)	50	miR139-5p
[17]	OSCC (24)<br>CONTROLS (24)	48	ADAM9
			Cathepsin V
			Kallikrein 5
			Kallikrein 7
[41]	OSCC (21)<br>CONTROLS (11)	32	miR- 412-3p
			miR-512-3p
			miR-302b-3p
			miR-517b-3p
[15]	OSCC (15)<br>OPMD (15)<br>CONTROLS (15)	45	Chemerin
			MMP-9
[56]	OSCC (30)<br>OPMD (30)<br>CONTROLS (40)	100	Vitamin C
[46]	OPMD (42)<br>OSCC (10)	52	Ornithine
			Carnitine
			Arginine
			o- Hydroxybenzoate
			N-acetylglucosamine -1 phosphate
			Ornithine
[47]	OSCC (20)<br>OPMD (20)	60	TSA

[47]	OSCC (30)<br>CONTROLS (30)<br>OPMD (60)	120	LDH
[28]	OPMD (35)<br>CONTROLS (35)	105	IL-6
			IL-8
[19]	OSCC (86)<br>CONTROLS (35)	111	AKR1B10
[13]	OPMD (58)<br>CONTROLS (65)	123	MMP-2
			MMP-8
			MMP-9
			TIMP-1
			TIMP-2
[29]	CONTROLS (24)<br>OSCC (41)	65	Eotaxin
			IFN-y
			IL-1β
			IL-6
			IL-8
			MIP-1b
			GRO
			TNF-α
			Eotaxin
[23]	OSCC (26)<br>CONTROLS (10)	36	ErbB2
			CEA
			Survivin
[48]	OSCC (101)<br>OPMD (58)<br>CONTROLS (35)	194	Glycine
			Proline
[57]	CONTROLS (20)<br>OPMD (20)<br>OSCC (20)	60	CRP

[58]	OSCC (33)<br>CONTROLS (34)	67	NAB2 mRNA
			CYP27A1 mRNA
			MAOB mRNA
			SIAE mRNA
			COL3A1 mRNA
			NPIPB4 mRNA
[59]	OSCC (30)<br>CONTROLS (30)	60	MMP-9
[24]	OSCC (30)<br>OPMD (10)<br>CONTROLS (10)	50	CD44v
			SYNE1
			miR-34a
[22]	OSCC (27)<br>CONTROLS (26)	53	ErbB2
			CEA
[21]	OSCC (32)<br>CONTROLS (37)	69	LDH
[60]	OSCC (30)<br>OPMD (30)<br>CONTROLS (30)	90	S100A2
			SLC3A2
			IL1RN
[61]	OSCC (116)<br>CONTROLS(65)	181	KPNA2
[51]	OSCC (63)<br>CONTROLS (60)<br>OPMD (21)	123	EGFR
[25]	OSCC (112)<br>OPMD (30)<br>CONTROLS (60)	202	Naa10p
			CEA
[32]	OSCC (21)	21	IL-1β
			IP-10
			MIP-1β
			VEGF

			IL-6
			IFN-γ
			IL-5
			IL-8
[62]	OSCC (30)<br>OPMD (25)<br>CONTROLS (30)	85	L-Fructose
[63]	OSCC (30)<br>OPMD (25)<br>CONTROLS (30)	35	CYFRA21-1
[33]	OSCC (71)	71	IL-17A
			IL-17F
			TNF-α
[64]	OSCC (30)<br>CONTROLS (30)	60	MMP-1
			PADI1
			TNC
			CSTA
			MMP-3
[36]	OSCC (16)<br>CONTROLS (8)	24	ANG
			ANG2
			HGF
			PIGF
			VEGF
			MMP-1
			MMP-2
			MMP-3
			MMP-8
			TIMP-1
			TIMP-2
[38]		96	PBSA

	OSCC (32)<br>TOBACCO CHEWERS (32)<br>CONTROLS (32)		FSA
[42]	OSCC (49)<br>CONTROLS (14)	63	miR-24-3p
[65]	OSCC (25)<br>OPMD (25)<br>CONTROLS (25)	75	β2-microglobulin
[66]	OSCC (60)<br>CONTROLS (20)	80	Cathepsin B
[37]	OSCC (41)<br>CONTROLS (10)	51	NUS1<br>RCN1
[67]	OSCC (15)<br>OPMD (15)<br>CONTROLS (15)	45	CD44<br>S100A7<br>S100P
[68]	OSCC (153)	153	MMP-13
[14]	OSCC (24)<br>OPMD (42)<br>CONTROLS (22)	88	MMP-9
[69]	OSCC (30)<br>OPMD (30)<br>CONTROLS (30)	90	8-OHdG
[70]	OSCC (33)<br>CONTROLS (12)	45	miR-30c-5p
[71]	OSCC (20)<br>OPMD (40)<br>CONTROLS (20)	80	KLK5
[26]	OSCC (66)<br>OPMD(66)<br>CONTROLS (20)	152	IL-1α<br>IL-6<br>IL-8

			IP-10
			MCP-1
			TNF-α
			HCC-1
			PF-4
[72]	OSCC (25)<br>OPMD(25)<br>CONTROLS (25)	75	LDH
[20]	OSSC (30)<br>OSMF (30)<br>CONTROLS (30)<br>TOBACCO<br>CHEWERS (30)	120	LDH

OSCC = oral squamous cell carcinoma; OPMD = oral potentially malignant disorder; OSMF = oral submucous fibrosis, .

Supplementary Table S2. Main findings from each study.

Reference	Molecule	Expression	AUC	P value	Sens	Spec
[44]	miR-21	↑ OSCC, ↑ OPMD	-	$p<0.001$	-	-
	miR-184	↑ OSCC, ↑ OPMD	-	$p<0.001$	-	-
	miR-145	↓ OSCC, ↓ OPMD	-	-	-	-
[43]	miR-21	↑ OSCC, ↑ OPMD	-	$p=0.003$	-	-
	miR-31	↑ OSCC, ↑ OPMD	-	$p<0.001$	-	-
[50]	CYFRA-21	↑ OSCC	-	$p<0.003$	88%	78%
[30]	SAT mRNA	↑ OSCC	0.799	$p=0.002$	-	-
	OAZ mRNA	↑ OSCC	0.799	$p=0.002$	-	-
	IL-1β mRNA	↑ OSCC	0.799	$p=0.002$	-	-
	IL-8 mRNA	↑ OSCC	0.799	$p=0.002$	-	-
[31]	IL-1β	↑ OSCC	0.9	$p<0.05$	-	-
	IL-8	↑ OSCC	0.9	$p<0.05$	-	-
	LGALS3BP	↑ OSCC, ↑ OPMD	0,76	$p<0.05$	-	-
[40]	miR-let-7a-5p	↑ OSCC	0.85	-	-	-
	miR-3928	↑ OSCC	0.074	-	-	-
	miR-7703	-	-	-	-	-
	miR-345-5p	-	-	-	-	-
	miR-1470	-	-	-	-	-
[52]	CD44	-	-	$P=0.06$	-	-
[34]	TNF-a	↑ OSCC, ↑ OPMD	-	$p<0.001$	95%	96%
[16]	CYFRA 21-1	↑ OSCC, ↑ OPMD	0.994	-	90%	97%
	LDH	↑ OSCC, ↑ OPMD	-	$p<0.0005$	-	-
	AMILASE	↑ OSCC, ↑ OPMD	-	$p<0.0005$	-	-
[53]	IL-4	↑ OSCC	-	$p=0.589$	-	-
	IL-10	↑ OSCC	-	$p=0.004$	-	-
	IL-13	↑ OSCC	-	$p=0.01$	-	-

	IL-1RA	↑ OSCC	-	p=0.96	-	-
[35]	GPx	↑ OSCC	-	p <0.05	-	-
	MDA	↑ OSCC	-	p <0.05	-	-
	TNF-α	↑ OSCC	-	p <0.05	-	-
	AFP	↑ OSCC	-	p <0.05	-	-
[54]	Transgeline mRNA	↑ OSCC	-	p<0.01	-	-
[14]	MMP9	↑ OSCC, ↑ OPMD	-	p<0.001	100%	54%
[18]	LDH	↑ OSCC	-	p<0.0009	-	-
[27]	H3F3A mRNA	-	0.66	p=0.0424	68%	35%
	IL-8 mRNA	↑ OSCC	0.43	p=4489	100%	87%
	IL-1β mRNA	-	0.6	p=0.1903	48%	20%
	DUSP1 mRNA	↑ OSCC	0.71	p=0.002	65%	23%
	OAZ1 mRNA	↑ OSCC	0.72	p=0.0035	85%	41%
	SAT1 mRNA	↑ OSCC	0.56	p=0.43	52%	27%
	S100P mRNA	↑ OSCC	0.58	p=0.3228	64%	39%
	TNF-α mRNA	↑ OSCC	0.968	-	-	-
	IL-6 mRNA	↑ OSCC	0.75	-	63%	10%
[55]	N-glycans	↑ OSCC	-	-	-	-
[45]	Sialic acid	↑ OSCC	-	p<0.01	-	-
[49]	miR-139-5p	↓ OSCC	0.8	p<0.006	-	-
[17]	ADAM9	↑ OSCC	0.7	-	-	-
	Cathepsin V	↑ OSCC	0.7	-	-	-
	Kallikrein 5	↑ OSCC	0.7	-	-	-
	Kallikrein 7	↑ OSCC	0.7	-	-	-
[41]	miR- 412-3p	↑ OSCC	0.871	-	-	-
	miR-512-3p	↑ OSCC	0.847	-	-	-
	miR-302b-3p	↑ OSCC	0.847	-	-	-
	miR-517b-3p	↑ OSCC	0.847	-		
[15]	Chemerin	↑ OSCC, ↑ OPMD	-	-	93%	80%
	MMP-9	↑ OSCC, ↑ OPMD	-	-	100%	93%

[56]	Vitamin C	↓ OSCC	-	-	-	-
[46]	Ornithine	↓ OSCC, ↓ OPMD	0.676	p<0.039	-	-
	Carnitine	↓ OSCC, ↓ OPMD	0.704	p<0.035	-	-
	Arginine	↓ OSCC, ↓ OPMD	0.689	p<0.015	-	-
	o- Hydroxybenzoate	↓ OSCC, ↓ OPMD	0.635	p<0.045	-	-
	N-acetylglucosamine -1 phosphate	↓ OSCC, ↓ OPMD	0.685	0.016	-	-
	R5P	↓ OSCC, ↓ OPMD	0.714	0.007	-	-
[47]	TSA	↑ OSCC	-	p<0.01	-	-
[20]	LDH	↑ OSCC	-	p<0.001	-	-
[28]	IL-6	↓ OSCC	-	p<0.001	-	-
	IL-8	↑ OSCC	-	p<0.0001	-	-
[19]	AKR1B10	↑ OSCC	-	p>0.01	-	-
[13]	MMP-2	↑ OSCC, ↑ OPMD	-	p=0.02	99%	86%
	MMP-9	↑ OSCC, ↑ OPMD	-	p=0.05	83%	50%
	MMP-8	↑ OSCC, ↑ OPMD	-	p=0.05	83%	67%
	TIMP-1	↑ OSCC, ↑ OPMD	-	p=0.09	67%	80%
	TIMp-2	↑ OSCC, ↑ OPMD	-	p=0.1	67%	8%
[29]	Eotaxin	↑ OSCC	0.662	p=0.030	71%	3%
	IFN-y	↑ OSCC	0.657	p=0.036	80.%	50%
	IL-1β	↑ OSCC	0.729	p=0.002	61%	79%
	IL-6	↑ OSCC	0.823	p<0.001	83%	71%
	IL-8	↑ OSCC	0.783	p=0.001	66%	79%
	MIP-1b	↑ OSCC	0.681	p=0.016	58%	79%
	GRO	↑ OSCC	-	p=0.078	-	-
	TNF-α	↑ OSCC	0.749	p=0.001	39%	100%
[23]	ErbB2	↑ OSCC	-	-	-	-
	CEA	↑ OSCC	-	p<0.05	-	-
	Survivin	↑ OSCC	-	p<0.05	-	-
[48]	Glycine	↓ OSCC	-	p<0.003	-	-

	Proline	↓ OSCC	-	p<0.005	-	-
[57]	CRP	↑ OSCC, ↑ OPMD	-	p<0.005	-	-
[58]	NAB2 mRNA	↓ OSCC	0.69	p=0.0023	-	-
	CYP27A1 mRNA	↓ OSCC	0.64	p=0.0016	-	-
	MAOB mRNA	↓ OSCC	0.63	P=0.0009	-	-
	SIAE mRNA	↓ OSCC	0.7	0.0370	-	-
	COL3A1 mRNA	↓ OSCC	0.67	0.0002	-	-
	NPIPB4 mRNA	↓ OSCC	0.64	0.0059	-	-
[59]	MMP-9	↑ OSCC	0.69	0,017	100%	27 %
[22]	ErbB2	↑ OSCC	-	p=0.1	-	-
	CEA	-	-	p=0.8	-	-
[21]	LDH	↑ OSCC	0.86	-	78%	78%
[24]	CD44v	↑ OSCC, ↑ OPMD	0.79	-	100%	60%
	SYNE1	↑ OSCC, ↑ OPMD	0.89	-	83%	100%
	miR-34a	↓ OSCC	-	-	-	-
[60]	S100A2	↑ OSCC, ↑ OPMD	0.89	-	83%	83%
	SLC3A2	↑ OSCC, ↑ OPMD	0.89	-	83%	83%
	IL1RN	↑ OSCC, ↑ OPMD	0.89	-	83%	83%
[61]	KPNA2	↑ OSCC	0.94	p<0.05	-	-
[51]	EGFR	↑ OSCC	0.68	p=0.0014	61%	69%
[25]	Naa10p	↑ OSCC, ↑ OPMD	0.88	p<0.05	-	-
	CEA	↑ OSCC, ↑ OPMD	0.85	p<0.05	-	-
[32]	IL-1β	↑ OSCC	-	p=0.049	-	-
	IP-10	↑ OSCC	-	p=0.047	-	-
	MIP-1β	↑ OSCC	-	p=0.033	-	-
	VEGF	↑ OSCC	-	p=0.014	-	-
	IL-6	↑ OSCC	-	p=0.005	-	-
	IFN-γ	↑ OSCC	-	p=0.036	-	-

	IL-5	↑ OSCC	-	p=0.048	-	-
	IL-8	↑ OSCC	-	p=0.004	-	-
[62]	L-fructose	↑ OSCC	-	P<0.0005	-	-
[50]	CYFRA 21-1	↑ OSCC	-	p=0.001	-	-
[33]	IL-17A	↑ OSCC	-	P< 0.001	-	-
	IL-17F	↑ OSCC	-	p<0.01	-	-
	TNF-α	↑ OSCC	-	p<0.01	-	-
[64]	MMP1	↑ OSCC	0.914	p <0.0001	-	-
	PADI1	↓ OSCC	0.827	p <0.0001	-	-
	TNC	↑ OSCC	0.813	p <0.0001	-	-
	CSTA	↓ OSCC	0.77	p <0.0001	-	-
	MMP3	↑ OSCC	0.753	p <0.0001	-	-
[36]	ANG	↑ OSCC	-	p=0.15	-	-
	ANG2	↑ OSCC	-	p=0.74	-	-
	HGF	↑ OSCC	-	p=0.22	-	-
	PIGF	↑ OSCC	-	p=0.089	-	-
	VEGF	↑ OSCC	-	p=0.97	-	-
	MMP1	↑ OSCC	-	p=0.35	-	-
	MMP2	↑ OSCC	-	p=0.83	-	-
	MMP3	↑ OSCC	-	p=0.28	-	-
	MMP8	↑ OSCC	-	p=0.1	-	-
	TIMP1	↑ OSCC	-	p=0.0063	-	-
	TIMP2	↑ OSCC	-	p=0.0063	-	-
[38]	PBSA	↑ OSCC	-	P < 0.05	-	-
	FSA	↑ OSCC	-	P < 0.05	-	-
[42]	miR-24-3p	↑ OSCC	0.738	P=0.02	-	-
[31]	LGALS 3bp0	↑ OSCC	0.7296	p=0.0008	-	-
	IL 1β	↑ OSCC	0.9017	p < 0.05	-	-
	IL-8	↑ OSCC	0.9017	p < 0.05	-	-
[65]	β2-microglobulin	↑ OSCC	-	P = 0.042	-	-

[66]	Cathepsin B	↑ OSCC	-	p< 0.001	-	-
[37]	NUS1	-	-	p=0.037	68.3%	70%
	RCN1	-	-	p=0.011	68.3%	90%
[67]	CD44	↑ OSCC	0.7	p=0.007	91%	54%
	S100A7	↑ OSCC	0.7	p=0.0296	81%	72%
	S100P	↑ OSCC	0.7	p=0.0296	81%	72%
[68]	MMP13	↑ OSCC	-	-	-	-
[14]	MMP9	↑ OSCC, ↑ OPMD	0.917	P<0.001	100%	59%
[69]	8-OHdG	-		<0.0001	-	-
[70]	miR-30c-5p	↑ OSCC	0,89	<0.001	80%	86%%
[71]	KLK5	↑ OSCC	0.853	<0.001	80%	77%
	uPA	↑ OSCC	0.821	<0.001	80%	73%
[26]	IL-8	↑ OSCC	0.842	<0.0001	76%	92%
	IL-1α	↑ OSCC	0.770	<0.001	67%	92%
	IL-6	↑ OSCC	0.921	<0.0001	82%	96%
	TNF-α	↑ OSCC	0.953	<0.0001	86%	100%
	IP-10	↓ OSCC	0.740	<0.001	64%	80%
[72]	LDH	↑ OSCC, ↑ OPMD	0,85	<0.001	76%	80%
[40]	miR- let -7a-5p	↓ OSCC	0,81	< 0.001	81%	73%
	miR- 3928	↓ OSCC		< 0.001	81%	73%

AUC = Area under the curve; Sens = Sensitivity; Spec = Specificity.
